# Impact of Differentiated Macrophage-Like Cells on the Transcriptional Toxicity Profile of CuO Nanoparticles in Co-Cultured Lung Epithelial Cells

**DOI:** 10.3390/ijms22095044

**Published:** 2021-05-10

**Authors:** Matthias Hufnagel, Ronja Neuberger, Johanna Wall, Martin Link, Alexandra Friesen, Andrea Hartwig

**Affiliations:** Department of Food Chemistry and Toxicology, Faculty of Chemistry and Biosciences, Institute of Applied Biosciences, Karlsruhe Institute of Technology (KIT), 76131 Karlsruhe, Germany; Matthias.Hufnagel@kit.edu (M.H.); Ronja.Neuberger@kit.edu (R.N.); Johanna.Wall@kit.edu (J.W.); Martin.Link@kit.edu (M.L.); Alexandra.Friesen@kit.edu (A.F.)

**Keywords:** nanotoxicology, co-culture, CuO, A549, THP-1, high-throughput RT-qPCR, gene expression profiles

## Abstract

To mimic more realistic lung tissue conditions, co-cultures of epithelial and immune cells are one comparatively easy-to-use option. To reveal the impact of immune cells on the mode of action (MoA) of CuO nanoparticles (NP) on epithelial cells, A549 cells as a model for epithelial cells have been cultured with or without differentiated THP-1 cells, as a model for macrophages. After 24 h of submerged incubation, cytotoxicity and transcriptional toxicity profiles were obtained and compared between the cell culture systems. Dose-dependent cytotoxicity was apparent starting from 8.0 µg/cm^2^ CuO NP. With regard to gene expression profiles, no differences between the cell models were observed concerning metal homeostasis, oxidative stress, and DNA damage, confirming the known MoA of CuO NP, i.e., endocytotic particle uptake, intracellular particle dissolution within lysosomes with subsequent metal ion deliberation, increased oxidative stress, and genotoxicity. However, applying a co-culture of epithelial and macrophage-like cells, CuO NP additionally provoked a pro-inflammatory response involving NLRP3 inflammasome and pro-inflammatory transcription factor activation. This study demonstrates that the application of this easy-to-use advanced in vitro model is able to extend the detection of cellular effects provoked by nanomaterials by an immunological response and emphasizes the use of such models to address a more comprehensive MoA.

## 1. Introduction

Nanomaterials are increasingly applied in various products due to their exceptional physicochemical properties. As a consequence, human exposure to nanomaterials is inevitable, emphasizing the need for appropriate tools for toxicological risk assessment [1]. Occupational exposure especially deserves special attention; in such contexts, inhalation is the most prevalent route of uptake [2]. Particles deposit in a size-dependent manner throughout the whole pulmonary system. While insoluble micro-sized particles can be cleared comparatively quickly and efficiently by the mucociliary escalator in the upper respiratory tract, clearance of insoluble nanoparticles in the lower respiratory is not as effective. This results in an extended retention half-life of these particles in the alveolar region, where they can interact with the pulmonary air-blood barrier [3]. This barrier consists of one layer of epithelial cells, dendritic cells inside and underneath the epithelium as well as macrophages; it is vascularized by capillaries underneath the barrier for optimized gas exchange. The epithelium itself consists of two different epithelia cell types, type 1 and type 2 (AT-I, AT-II). While AT-I covers most of the surface of the alveolar epithelium (>93%), AT-II cells cover less area (ca. 7%) but are more numerous (16% compared to 8% of cells, respectively, in the distal lung). Furthermore, AT-II cells serve for surfactant production and act as precursors for AT-I cells [4,5,6]. Also, AT-II cells have been shown to release several immune response-related chemokines, e.g., after an infection, resulting in the recruitment of innate immune cells, as well as pro-inflammatory cytokines [6]. One of the most frequently applied AT-II cell models is the adenocarcinoma cell line A549, which exerts important AT-II morphology and functionality, such as lamellar body formation, surfactant secretion at air–liquid interface cultivation, pro-inflammatory cytokine release, and AT-II typical transport properties [4,5,7]. Regarding xenobiotic metabolism, some contradictory results have been published. Thus, Foster and colleagues showed consistent metabolic properties of A549 cells when compared to type II pulmonary epithelial cells with respect to phase I oxidative metabolism [7]. However, Castell and colleagues describe the capacity of xenobiotic biotransformation via phase I metabolism as not comparable with normal lung tissue while phase II activity was found to be within the same range as lung tissue [8].

Due to ineffective particle clearance at higher doses, nanoparticles can interact not only with epithelial cells but also with cells of the immune system such as macrophages and dendritic cells. Therefore, for toxicological studies, it would be advantageous to use co-cultures of epithelia and immune cells to mimic in vivo conditions more realistically. Furthermore, such systems have been shown to allow for cell–cell communication [5]. Presently, advanced in vitro models have already been used in several studies investigating the adverse effects of nanomaterials. However, models with up to four cell lines are often very complex and also expensive due to the use of transwell inserts (e.g., [9,10,11,12]). One easy-to-use co-culture system to mimic the alveolar barrier is a co-culture consisting of an A549 cell, as a very well-known model for alveolar epithelia type II cells, together with differentiated THP-1 (dTHP-1) cells resembling macrophage-like cells [5]. Only a few particle toxicity studies have been performed applying this comparatively easy-to-use advanced in vitro model under submerged conditions, revealing, for instance, changes in inflammatory response upon particle exposure [13,14,15,16,17]. Within the present study, for the first time, we combined this co-culture model with a comprehensive gene expression analysis to potentially identify differences in the MoA between the cell models.

Since the toxicity of CuO nanoparticles (NP) after submerged [18,19] and air–liquid interface exposure [20,21] has been extensively studied and largely understood, this particle species was used to explore toxicity profiles under single- and co-culture conditions. Regarding the impact of CuO NP in A549 cells, the present study continues from previous studies of our working group, applying a high-throughput RT-qPCR (HT RT-qPCR) system to investigate the expression of 95 genes in 96 samples in parallel by using a custom-designed gene set focusing on the impairment of genomic stability. Briefly, the identified MoA included endocytotic particle uptake, intracellular particle dissolution within lysosomes, increased oxidative stress, and genotoxicity [22,23,24,25]. Within the present study, the gene set has been extended with markers for inflammation and fibrosis. Regarding the impact of CuO NP on the applied co-culture, only one study has been published so far investigating cell viability, reactive oxygen species (ROS) induction, and IL-8 secretion [26]. Here, only IL-8 secretion showed cell-type-dependent results.

Within the present study the impact of CuO NP on A549 cells in mono- and co-culture with dTHP-1 was elucidated using a comprehensive transcriptional analysis by the HT RT-qPCR approach described above. Major aim was to identify possible differences in the MoA on a transcriptional level of both cell models and to elucidate whether or not the combination of co-culture and HT RT-qPCR can be applied as an easy-to-use screening model for nanomaterial hazard identification. The results demonstrate quite a similar impact of both culture systems on genes related to xenobiotic metabolism, fibrosis, metal homeostasis, oxidative stress response, apoptosis and cell cycle regulation as well as DNA damage response and repair, but pronounced differences in transcriptional toxicity profiles for markers of the inflammatory response, thus demonstrating the applicability of the applied tools for the risk assessment of nanomaterials.

## 2. Results

### 2.1. Nanoparticle Characterization

Endotoxin levels of the CuO NP in double-distilled H_2_O were below the detection limit of 0.1 EU/mL. Physicochemical properties were characterized thoroughly either in previous projects or within this study regarding their primary particle size (transmission electron microscopy, TEM), hydrodynamic diameter, ζ-potential, specific surface area, and solubility in the cell culture medium after 24 h. These data are summarized in Table 1.

**Figure 1:**
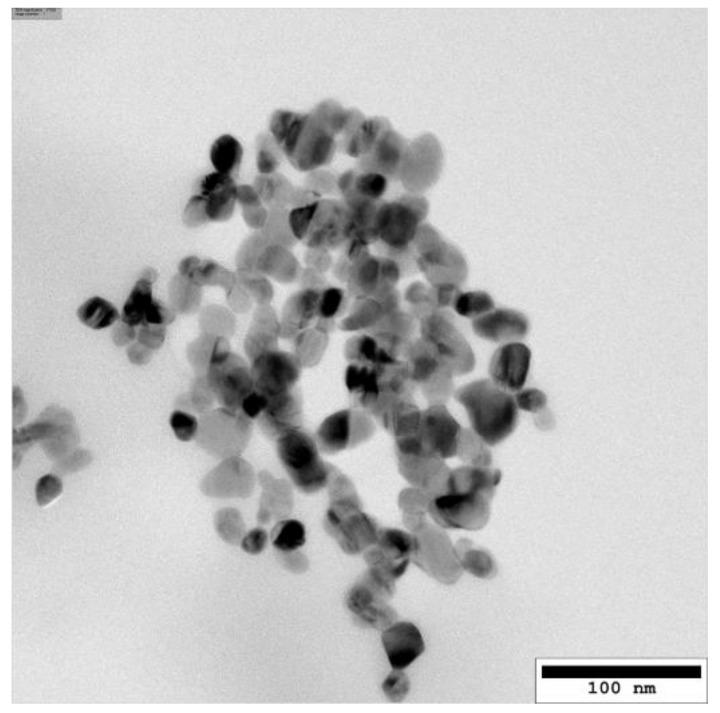
Representative TEM image of the CuO NP at 2.56 mg/mL in 0.05% bovine serum albumin (BSA) after dispersion. More images are shown in Supplementary Figure S1.

CuO particles were mostly spherical, relatively narrow distributed (10–40 nm), slightly aggregated, and revealed a primary particle size of 17.1 ± 0.4 nm (Figure 1). Using dynamic light scattering (DLS), a hydrodynamic diameter of 175 nm, a PDI of 0.49, and a ζ-potential of −14.8 mV in a 100 µg/mL suspension in serum-supplemented RPMI was obtained. The solubility of CuO NP in serum-supplemented RPMI was found to be 23 ± 12% after 24 h. A specific surface area (SSA) of 47 m^2^/g was determined within the Sustainable Nanotechnologies (SUN) project [27]. In addition, these particles were further characterized by Gosens and colleagues using x-ray diffraction (XRD) and x-ray photoelectron sprectroscopy (XPS) techniques, proving the presence of monoclinic CuO and the appearance of copper, oxygen, and carbon [28].

### 2.2. Cytotoxicity of CuO in Mono- and Co-Culture

**Figure 2:**
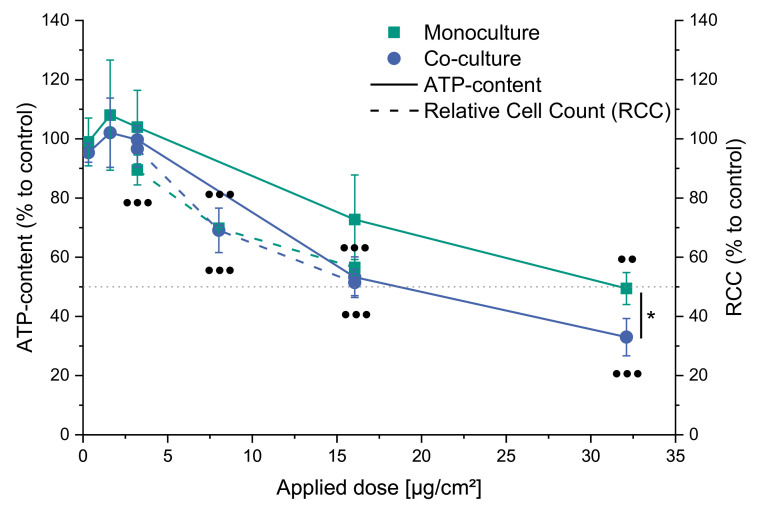
Impact of CuO NP on the ATP-content and relative cell count (RCC) of a mono- (A549) and co-culture (A549 + dTHP-1). Monocultures are displayed in green squares and co-culture in blue circles. ATP-content is shown as a solid line, and RCC is plotted as a dashed line. Shown are the mean values of three independent experiments ± standard deviation (SD). Statistics were performed using either *t*-test (* ≤ 0.05) to compare differences between mono- and co-culture or ANOVA-Dunnet’s *t*-test (● ≤ 0.05, ●● ≤ 0.01, ●●● ≤ 0.001) to compare differences between doses of one endpoint.

Within this study, the relative cell count (RCC) and the ATP content were chosen as parameters of cytotoxicity (Figure 2). In the first step, the ATP content was determined by applying five doses of CuO NP (0.32, 1.6, 3.2, 16.1, and 32.1 µg/cm^2^). Within both culture systems, dose-dependent cytotoxicity was observed, with a tendency towards higher sensitivity of the co-culture system, which was statistically significant at 32.1 µg/cm^2^. For example, ATP contents of 73 and 53% were observed after applying 16.1 µg/cm^2^ and 49 and 33% at 32.1 µg/cm^2^ in mono- and co-culture, respectively. Subsequently, three doses for each culture were chosen for RCC determination and transcriptional toxicity profiling, representing no/low, mid, and high (up to 50%) cytotoxicity. Since preliminary experiments revealed a higher sensitivity of RCC compared to the ATP-content, 3.2, 8.0, and 16.1 µg/cm^2^ were applied resulting in 89, 70, and 57% viability in the monoculture and 97, 69, and 51% viability in the co-culture, respectively. For this parameter of cytotoxicity, no relevant difference between the two cell culture models was apparent. To assess the potential role of copper ions released extracellularly from the CuO NP on the observed cytotoxicity, copper ion concentrations were determined to be about 150 µM at the highest dose, resulting in an RCC of 82 ± 2% and 80 ± 7% in the mono- and co-culture, respectively.

### 2.3. Transcriptional Toxicity Profiles in Mono- and Co-Culture

**Figure 3:**
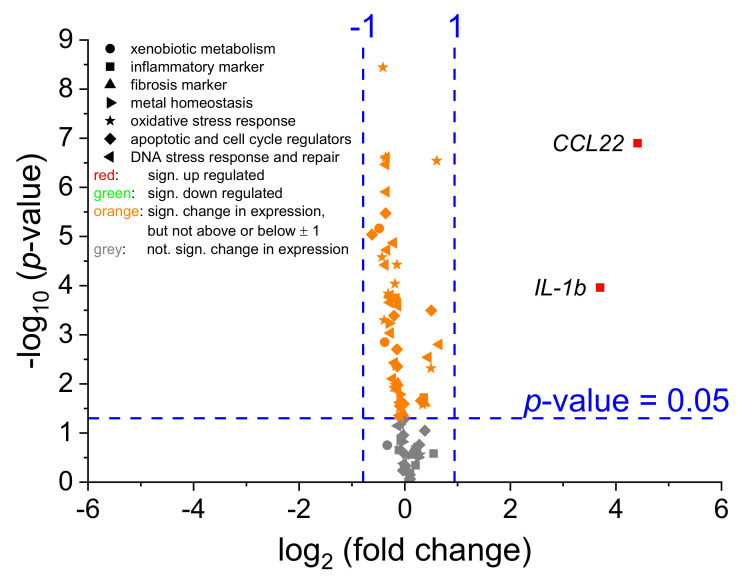
Differences in gene expression between untreated mono- (A549) and co-culture (A549 + dTHP-1) using a high-throughput RT-qPCR system. The volcano plot displays the extent (log_2_ (fold change), *x*-axis) and the statistical significance (−log_10_ (*p*-value), *y*-axis) of gene expression alterations between the co-culture compared to the monoculture. Vertical lines indicate a relevant expression change of 50% reduction (−1) or doubling (+1). The horizontal line displays the statistical significance threshold (*p* ≤ 0.05, *n* = 3, independent samples *t*-test). Red: significant up-regulated genes. Green: Significant down-regulated genes. Orange: significant changes in gene expression, but not considered relevant (−1 ≤ x ≤ 1). Grey: no significant change in gene expression.

To obtain transcriptional toxicity profiles, a high-throughput RT-qPCR system was applied investigating 95 genes in 96 samples in parallel, comprising markers related to xenobiotic metabolism, metal homeostasis, oxidative stress response, apoptosis, and cell cycle regulation, as well as DNA damage response and repair [25], supplemented with markers of inflammation and fibrosis (Supplementary Table S1). In a first step, basal gene expression profiles of mono- and co-cultures were analyzed and referred to the untreated monoculture control. To assess changes in gene expression profiles, two parameters were analyzed. First, alterations in gene expression were evaluated with respect to statistical significance. Since even minor alterations appear to be significant when error bars are small, as a second parameter, changes were judged based on fold-changes of expression levels of the respective genes. Here, a reduction of at least 50% (log_2_-fold change ≤ −1) or a doubling (log_2_-fold change ≥ 1) when compared to the respective control were considered relevant [23]. The comparison of basal gene expression of both culture models is displayed as a volcano plot in Figure 3. Even though significant differences (expression changes −1 ≤ x ≤ 1) were observed for several genes, a relevant—according to our criteria as outlined above—up-regulation (≥1 log_2_-fold change) in the co-culture when compared to the monoculture was observed for only two genes, namely *CCL22* (6.9 log_2_-fold change, 119.4-fold induction), coding for a macrophage-derived chemokine (MDC, CCL22), and *IL-1b* (4.0 log_2_-fold change, 16-fold induction), coding for a pro-inflammatory cytokine of the IL1-family (IL-1β). A comprehensive list of all analyzed genes including their log_2_-fold change and *p*-values is provided in Supplementary Table S2.

**Figure 4:**
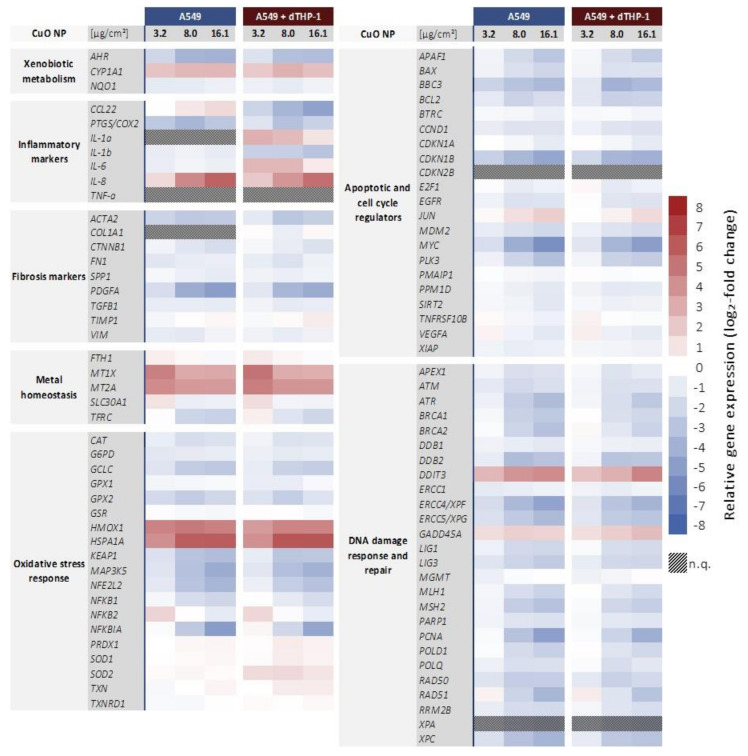
Overview of the impact of CuO NP on A549 cells cultured in monoculture or in co-culture with dTHP-1 cells using a high-throughput RT-qPCR approach with a custom-designed gene set. The genes under investigation have been clustered into groups associated with xenobiotic metabolism, inflammation, fibrosis, metal homeostasis, oxidative stress response, apoptosis, and cell cycle regulation as well as DNA damage response and repair. Both cell models were treated with CuO NP for 24 h. Displayed are the log_2_-fold changes of relative gene expression as a heatmap. Red colors indicate an enhanced expression, and blue colors indicate a down-regulation. Shown are the mean values of at least three independently conducted experiments. n.q.: not quantifiable due to low expression levels.

Subsequently, the impact of CuO NP on gene expression profiles was examined in both cell culture models after 24 h incubation with three doses (3.2, 8.0, and 16.1 µg/cm^2^). An overview after treatment with CuO NP of the mono- and co-culture, respectively, is displayed as a heatmap in Figure 4. On the first view, both cell models revealed similar toxicity profiles, with the exception of alterations in the inflammatory response. The data are also summarized as x-fold changes in Supplementary Table S3. Main observations in both cell models for each investigated gene cluster are described in more detail in the following sections.

#### 2.3.1. Impact on Genes Related to Inflammation and Fibrosis

**Figure 5:**
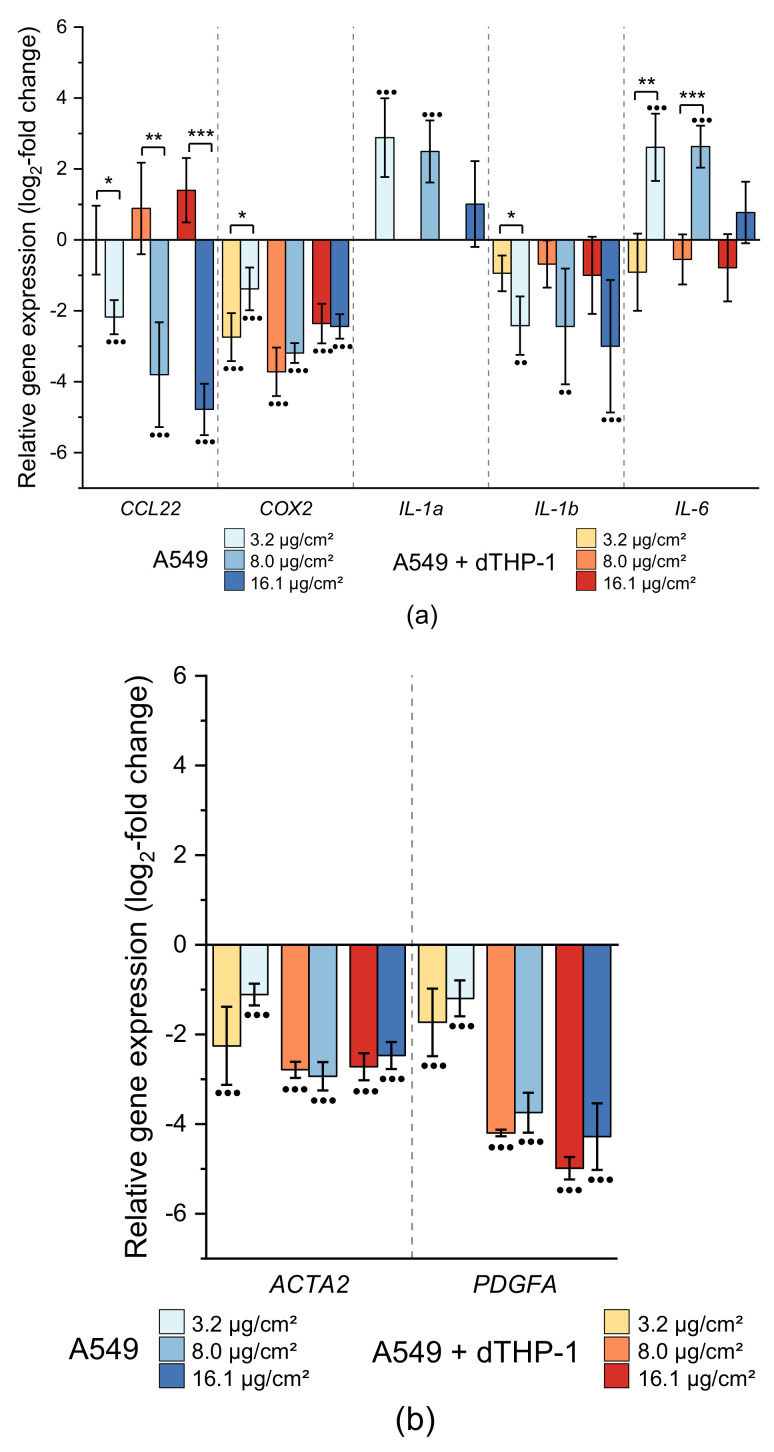
Impact of CuO NP on genes related to inflammation and fibrosis in A549 monoculture (A549) and co-culture (A549+dTHP-1) after 24 h incubation. (**a**) Gene expression of inflammatory markers; (**b**) gene expression of fibrotic markers. Depicted are the log_2_-fold changes of at least three independently conducted experiments ± SD. Significantly different from negative controls: ● ≤ 0.05, ●● ≤ 0.01, ●●● ≤ 0.001 (ANOVA-Dunnett’s *t*-test); significantly different between cell models: * ≤ 0.05, ** ≤ 0.01, *** ≤ 0.001 (unpaired *t*-test).

With regard to inflammatory markers, several genes revealed a cell culture model-dependent response (Figure 5A). Here, both, down-regulation (*CCL2*, *COX2*, *IL-1b*) and up-regulation (*IL-1a*, *IL-6*) were observed after CuO NP exposure. In particular, *CCL22* was dose-dependently depressed down to −4.8 log_2_-fold (−27.9-fold) in the co-culture, while in monoculture, an increased expression up to 1.4 log_2_-fold (2.6-fold) was apparent. For this gene, the cell-culture-dependent difference was statistically significant at each applied dose. In addition, a pronounced down-regulation of *COX2*, coding for cyclooxygenase 2 and representing an inflammation marker, within both cell cultures was observed, at a nearly constant level across all applied doses. Furthermore, *IL-1b* expression was constantly down-regulated across all applied doses in both cell models down to −0.9 and −3.0 log_2_-fold (−1.9 and −8 fold), respectively. Even though effects were more pronounced in the co-culture system, only the difference at the lowest dose reached statistical significance between the cell models due to comparatively high standard deviations. Marked differences were seen for *IL-6*: while the expression in monoculture was slightly, but consistently, decreased down to −0.9 log_2_-fold (−1.9-fold) across all applied doses, the co-culture responded with an up to 2.6 log_2_-fold (6-fold) induction at 3.1 and 8.0 µg/cm^2^ CuO. *IL-1a* expression was only quantifiable in the co-culture revealing an enhanced expression of 2.9 log_2_-fold (7.5-fold) at the lowest dose, with lower induction levels at higher doses. Finally, *IL-8* was induced dose-dependently in both cell models up to 5.6 log_2_-fold (48.5-fold) without any difference between mono- and co-culture (Figure 4).

In the context of fibrosis markers, a consistent down-regulation of several genes across all applied doses was apparent. The strongest depressions were observed for *ACTA2* and *PDGFA*, coding for alpha smooth muscle actin (α-SMA) and a growth factor of the PDGF family, respectively. While *ACTA2* was consistently down-regulated roughly −3 log_2_-fold (−8 fold), *PDGFA* was dose-dependent depressed down to −5 log_2_-fold (−32-fold). However, no difference was seen between mono- and co-culture (Figure 5). Furthermore, in the case of *COL1A1*, quantification was only possible in the co-culture since expression levels in A549 cells themselves were below the detection level. For all other markers, at most slight differences were apparent, which were not considered relevant.

#### 2.3.2. Impact on Genes Related to Xenobiotic Metabolism and Metal Homeostasis

Regarding xenobiotic metabolism, two genes were found to be affected by CuO, independent of the applied cell culture system (Figure 4). Here, a −2 log_2_-fold (−4-fold) down-regulation of *AHR* was apparent at the lowest dose of 3.2 µg/cm^2^, reaching a constant expression level of −3.8 log_2_-fold (−13.9-fold) at higher doses. In contrast, the expression of *CYP1A1* was increased at all doses applied from 2.2 to 2.6 log_2_-fold (4.6 to 6-fold).

**Figure 6:**
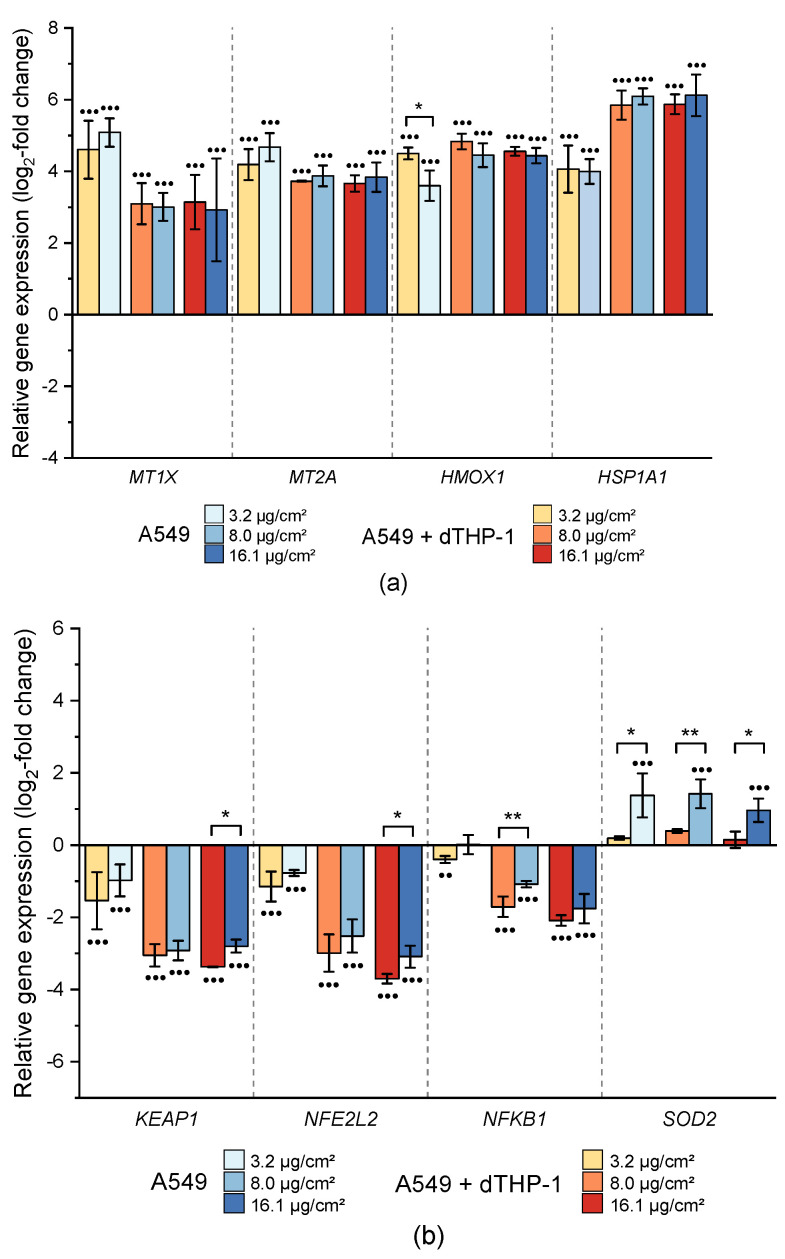
Impact of CuO NP on genes related to metal homeostasis and oxidative stress response in A549 monoculture (A549) and co-culture (A549+dTHP-1) after 24 h incubation. (**a**) Expression of genes related to metal homeostasis and transcriptional oxidative stress markers; (**b**) expression of genes associated with anti-oxidative defense mechanisms. Depicted is the log_2_-fold change of at least three independently conducted experiments ± SD. Significantly different from negative controls: ● ≤ 0.05, ●● ≤ 0.01, ●●● ≤ 0.001 (ANOVA-Dunnett’s *t*-test); significantly different between cell models: * ≤ 0.05, ** ≤ 0.01, *** ≤ 0.001 (unpaired *t*-test).

*MT1X* and *MT2A*, coding for metallothioneins and thus related to metal homeostasis, revealed an increased expression level after CuO NP exposure in both cell models (Figure 6A). In both cases, the lowest dose provoked the most pronounced induction by 4.6 and 5.1 log_2_-fold (24.3 and 34.3-fold) for *MT1X* and 4.2 and 4.7 log_2_-fold (18.4 and 26-fold) for *MT2A*, respectively. With increasing dose, a plateau in gene expression was observed for both *MT1X* and *MT2A*. Altogether, no statistically significant differences between mono- and co-culture were apparent in these gene clusters.

#### 2.3.3. Impact on Genes Related to Oxidative Stress Response

As a broad picture, an impact on the transcriptional oxidative stress response after CuO NP exposure was apparent from an up-regulation of oxidative stress markers (Figure 6a) and a down-regulation of several genes associated with anti-oxidative defense mechanisms (Figure 6b). Most pronounced was an up-regulation observed for *HMOX1* and *HSPA1A*, coding for heme oxygenase 1, and heat shock protein A1A, respectively. *HMOX1* expression was consistently induced up to 4.8 log_2_-fold (28-fold) in both cell culture models, with a small but statistically significant lower induction in the co-culture at the lowest dose. Regarding *HSPA1A*, an almost identical induction in mono- and co-culture was observed, reaching a maximum of 6 log_2_-fold (64-fold) expression level. In addition, *SOD2*, coding for manganese-dependent superoxide dismutase 2, was up-regulated. While the induction was small and not considered relevant in the monoculture, the effect was more pronounced in the co-culture system, where a constant induction of 1.4 to 1.6 log_2_-fold (2.6 to 3-fold) was observed. Most other genes associated with anti-oxidative defense mechanisms were down-regulated; effects were either dose-independent (*CAT*, *G6PD*, *GPX2*) or dose-dependent (*GCLC*, *KEAP1*, *MAP3K5*, *NFE2L2*, *NFKB1*, *NFKBIA*), after CuO NP exposure (Figure 4). When comparing the two cell culture models, *NFKB1*, *KEAP1*, and *NFE2L2* exerted slightly lower effects in the co-culture system as compared to the A549 monoculture (Figure 6b).

#### 2.3.4. Impairment of Genes Related to Apoptosis and Cell Cycle Regulation

Considering genes related to apoptosis and cell cycle regulation, all genes except for one (*JUN*) were down-regulated, most of them independently from the applied cell culture system. In particular, a distinct dose-dependent down-regulation of *BBC3*, *CDKN1B*, *MYC*, and *PLK3* was apparent (Figure 4). Only three genes showed rather slight but at some concentrations statistically significant changes between the cell models, namely *BAX*, *PPM1D,* and *SIRT2* (Supplementary Figure S2). However, these differences did not seem to be relevant since similar expression patterns with similar degrees of down-regulation were observed across all applied doses.

#### 2.3.5. Impact on Genes Related to DNA Damage Response and Repair

**Figure 7:**
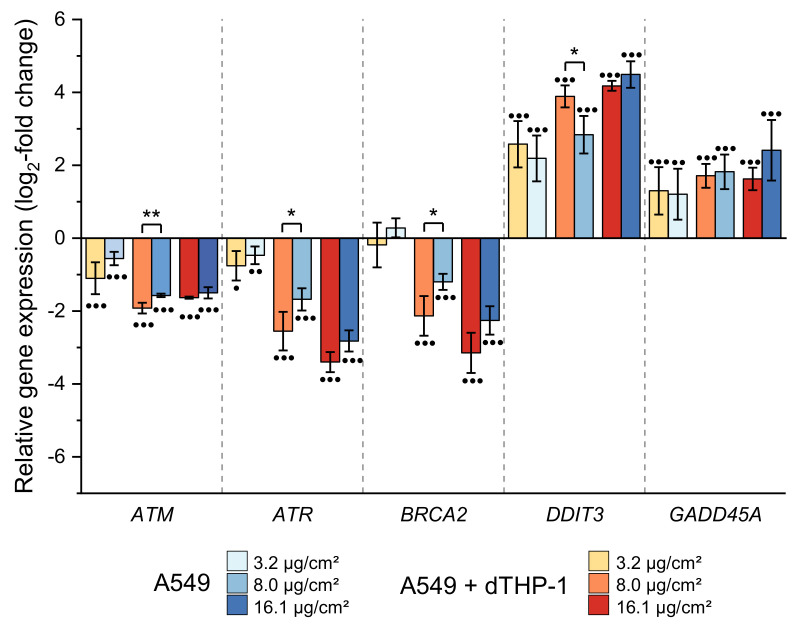
Impact of CuO NP on genes related to DNA damage response and repair in A549 monoculture (A549) and co-culture (A549+dTHP-1) after 24 h incubation. Depicted is the log_2_-fold change of at least three independently conducted experiments ± SD. Significantly different from negative controls: ● ≤ 0.05, ●● ≤ 0.01, ●●● ≤ 0.001 (ANOVA-Dunnett’s *t*-test); significantly different between cell models: * ≤ 0.05, ** ≤ 0.01, *** ≤ 0.001 (unpaired *t*-test).

CuO NP provoked a pronounced impact on various genes associated with DNA damage response and repair. Most strikingly, genes coding for DNA damage response proteins (*DDIT3*, *GADD45A*) were induced, whereas genes coding for specific DNA repair proteins were decreased in their expression. Thus, the genotoxic stress marker *DDIT3* showed a dose-dependent increase up to 4.5 log_2_-fold (22.6-fold), with both cell culture systems showing a similar induction (Figure 7). In addition, *GADD45A*, as a second marker for genotoxic stress, was consistently induced in both cell systems across all applied doses (Figure 7). Nevertheless, genes coding for specific DNA damage signaling or DNA repair factors such as *ATR*, *ATM*, *BRCA2*, *ERCC4*, *ERCC5*, or *PCNA* exerted a dose-dependent down-regulation. Even though the pattern was similar in both cell culture systems, in the case of *ATR*, *ATM,* and *BRCA2*, there was a tendency towards stronger effects in the monoculture as compared to the co-culture system, which was statistically significant at some dose points.

## 3. Discussion

Within this study, CuO NP were applied on A549 cells in mono- and co-culture with dTHP-1 cells followed by a comprehensive transcriptional analysis using an HT RT-qPCR approach and applying a custom-designed gene set as described previously [22,23,24,25]. For the purpose of this study, the gene set originally comprising markers of metal homeostasis, (oxidative) stress response, DNA damage and repair, cell cycle control, and apoptosis was extended with genes coding for inflammatory and fibrotic pathways (Supplementary Table S1). The aim of the present investigation was to identify potential differences in the MoA on a transcriptional level of both cell models and to elucidate whether the combination of co-culture and HT RT-qPCR can be applied as an easy-to-use screening model for nanomaterial hazard identification. Since no separation of epithelial cells and macrophage-like cells was performed within the co-culture experiments and A549 were more numerous, most effects were likely dominated by A549 cells, potentially influenced by dTHP-1 cells in the co-culture system.

The cytotoxic effects of CuO NP are consistent with previous studies using A549 cells (e.g., [18,19]). When comparing the two cell models, a tendency towards higher toxicity of CuO in the co-culture system was observed when assessing the ATP-content, while for RCC, a similar toxicity was observed. This is in general agreement with data from literature using A549 in mono- or co-culture, with one study reporting a slight difference [13], while others did not observe any difference [26,29]. To elucidate the contribution of copper ions to the observed toxicity, the solubility of the applied particles after dispersion for 24 h in cell culture medium was determined. Here, 23% solubility was observed within the cell culture medium applied in the present study, which is considerably lower than 67% previously found in another cell culture medium (DMEM) [30], indicating a moderately dependent solubility. The soluble fraction of 23% reflects a concentration of roughly 150 µM copper ions at the highest applied dose for gene expression and RCC. As previously shown within our working group, CuCl_2_ reduced the colony-forming ability in A549 cells by 50% at 252 µM [18], while no relevant impact by CuCl_2_ was apparent on RCC in BEAS-2B cells [22]. In experiments performed within the present investigations, 150 µM CuCl_2_ corresponding to 25% solubility resulted in roughly 80% RCC in both cell systems, indicating that most cytotoxicity resulted from CuO NP, with a small impact of extracellularly released copper ions. With regard to the potential contribution of soluble copper ions to the gene expression alterations, Strauch and colleagues previously showed that a copper ion concentration of 126 µM did provoke some alterations on the transcriptional level [22]. Accordingly, it is suggested that the highest possibly released extracellular concentration of 150 µM water-soluble copper ions might have an impact on the observed gene expression changes within the present study. However, it was also shown that the endocytotic uptake and lysosomal acidification add significantly to intracellular copper levels and transcriptional changes provoked by CuO NP [24]; therefore, it is suggested that extracellular copper ions contribute to the cellular effects investigated but are not the driving force.

In order to obtain information on different gene expression patterns in between the two cell culture models, in a first step, untreated controls of both cultures were analyzed and compared. Statistically significant and, according to our criteria, relevant (±1 log_2_-fold) changes were seen only for two genes, namely an increased expression of up to almost 120-fold in the case of *CCL22* and 16-fold in the case of *IL-1b* in the co-culture system when compared to the monoculture of A549. *CCL22* encodes the protein macrophage-derived chemokine (MDC, CCL22) which is mainly synthesized by macrophages and dendritic cells [31], explaining the enhanced expression in the co-culture with macrophage-like cells. *IL-1b* and *IL-1a* both encode pro-inflammatory cytokines of the IL1-family (IL-1β and IL-1α), which are produced by multiple cells, including macrophages [32,33]. In contrast to *IL-1b*, the expression of *IL-1a* was only quantifiable in the co-culture, indicating its induction. Kolesar and colleagues previously characterized the expression of cytokine genes in THP-1 cells after co-culture with A549 cells. Among other cytokines, a strong increase in *IL-1b* expression in THP-1 cells was observed after 24 h, indicating an impact of the epithelial cells on gene expression in macrophages in their co-culture system [34]. Furthermore, both *CCL22* and *IL-1b* are markers for macrophage polarization either towards the pro-inflammatory M1 (*IL-1b*) or the anti-inflammatory M2 (*CCL22*) phenotype [35]. The simultaneous expression of these genes within this study suggests a mixture of M1 and M2 macrophages after differentiation of THP-1 cells.

Transcriptional toxicity profiles of CuO NP in A549 cells have already been obtained previously after submerged and air-liquid exposure conditions [22,23,24]. The presented data within this study for A549 cells are consistent with the proposed “Trojan horse type mechanism”. Briefly, this involves endocytic particle uptake followed by lysosomal degradation of the NP with subsequent copper ion release; this is in agreement with the enhanced *MT1A* and *MT2X* expression. This leads to elevated levels of intracellular copper ions, which induce an oxidative (*HMOX1*, *HSPA1A*) and inflammatory (*IL8*) response via the activation of redox-sensitive transcription factors such as Nrf2, HSF-1, NF-κB, and AP-1. In support of this theory, within the present study, *HMOX1* and *HSP1A1* were up-regulated in a pronounced manner. Nevertheless, other genes involved in the anti-oxidative stress response such as *GPX2*, *GSR*, *KEAP1*, *NFE2L2*, and *NFKB1* were downregulated. This may be explained by the fact that, in A549 cells, Nrf2 is constitutively activated due to a dysfunction of its negative inhibitor Keap1 [36]. A similar down-regulation of several anti-oxidative genes in A549 cells was also observed in a previous study in our working group, while the complete oxidative stress response was apparent in Beas-2B cells [22]. Regarding the role of Nrf2 in *HMOX1* transcription, it is known that not only Nrf2 but also members of the heat-shock factor, NF-κB, and AP-1 families regulate *HMOX1* expression [37], which may explain the induction of *HMOX1* expression in A549 cells. Interestingly, *HMOX1* expression is also even more pronounced in Beas-2B cells [22]. Nevertheless, several Nrf2- independent genes associated with apoptosis and cell cycle regulation, such as *APAF1*, *BAX*, *BBC3*, *BCL2*, *CCND1*, *CDKN1B*, *MDM2*, *MYC*, and *PLK3*, were clearly down-regulated as well. One potential explanation is epigenetic alterations as postulated by Chibber and Shanker [38]; however, this mechanism would need to be further investigated. The induction of the genotoxic stress markers *DDIT3* and *GADD45A* is in agreement with the induction of DNA damage by elevated levels of redox-active copper ions; however, at the same time, other genes related to DNA damage signaling such as *ATM* and *ATR* as well as genes coding for specific DNA repair pathways were consistently down-regulated. These observations resemble those made in our previous study under submerged conditions [22]. The repression of DNA repair pathways despite the induction of DNA damage is still not fully understood; however, Collin and colleagues suggested that cellular senescence may result in repressed DNA repair genes [39]. An induction of senescence was, so far, shown for copper sulfate in HeLa cells and for Cu NP in *Enchytraeus crypticus* [40,41]; a potential induction of senescence by CuO NP in mammalian cells remains to be elucidated.

Regarding xenobiotic metabolism, within the present study, *AHR* was down-regulated while *CYP1A1* was up-regulated. The latter is mainly regulated by aryl hydrocarbon receptor (AhR). Concerning literature data, the activation of AhR by Cu^2+^ has been described so far for rat cells at low copper ion concentrations (30 µM), while at higher concentrations, both *CYP1A1* and *AHR* expression was down-regulated, possibly due to increased oxidative stress [42]. This aspect needs to be further investigated.

Considering the differences between the two cell culture models within the gene clusters described above and identified previously contributing to CuO NP transcriptional toxicity profiles, some statistically significant results were apparent. However, these changes were only slight and/or only apparent at single doses and therefore not considered relevant. This concerns the expression of *ATM*, *BAX*, *COX2*, *FN1*, *GPX1*, *GSR*, *HMOX1*, *KEAP1*, *NFE2L2*, *NFKB1*, *PMAIP1*, *PRDX1*, *PPM1D*, *SOD1*, *SIRT2*, and *TGFB1*. In contrast, changes in the expression of *ATR*, *BRCA2*, and *SOD2* were more distinct between the two cell culture systems. While *BRCA2* and *ATR* were more pronouncedly down-regulated in A549 cells when compared to the co-culture system, *SOD2* was up-regulated in the co-culture, and no induction was obvious in A549 cells alone. *SOD2* up-regulation is linked to a NF-κB activation [43]; however, other target NF-κB genes such as *BAX*, *BCL2*, *COX2*, *NFKBIA*, and *NFKB1* [44,45,46,47] were down-regulated. Therefore, no distinct conclusion regarding NF-κB activation in the co-culture system can be made.

As already stated, the previously applied gene set [22,23] was complemented by fibrotic and inflammatory markers within this study. Considering fibrosis, a down-regulation of respective marker genes was generally apparent. *ACTA2*, coding for the epithelial–mesenchymal transition (EMT) marker α-SMA [48], and *PDGFA*, coding for the fibrosis marker protein platelet-derived growth factor subunit A [49], were most affected by CuO NP. The only difference between the two cell systems within the fibrotic gene cluster was the up-regulation of *COL1A1*, coding for the mesenchymal marker collagen type 1A1 [50]. This observation was rather surprising, as CuO NP provoke oxidative stress, and ROS has been suggested to cause EMT, and thus the onset of fibrosis [51].

However, the most distinct differences in the transcriptional toxicity profile of CuO NP between the cell systems were observed in the gene cluster of inflammatory markers. Besides the induction of *IL6* and *IL-1a,* a down-regulation of *CCL22* and *IL-1b* was apparent only in the co-culture. In contrast, both cell systems revealed an increased *IL-8* expression, in contrast to the observation of Wand and colleagues, who observed a cell-system-specific *IL-8* response [26]. All of these genes are cellular markers for a pro-inflammatory response [52,53,54]; therefore, the up-regulation of these genes indicates an inflammatory potential of CuO NP. However, the dose-dependent down-regulation of *CCL22* and *IL-1b* does not match this scenario. The reason behind this observation might be the increased cytotoxicity of CuO NP in dTHP-1 cells in the co-culture such that less dTHP-1 originated mRNA in the gene expression analyses. This is supported by the observation of a strong induction of these two genes in the untreated co-culture compared to the A549 monoculture. In addition, this could also explain the repression of *IL-1a* and *IL-6* at the highest applied dose that was investigated. Regarding the proteins encoded by *IL-1a* and *IL-1b* (IL-1α, IL-1β), both are part of the NLRP3 inflammasome response, which is induced by ROS [53,55]. Moreover, the respective proteins of *IL-6* and *IL-8* are induced by several transcription factors such as AP-1, NF-κB, STAT3, and C/EBPβ [56,57,58]. All of these genes have been implicated in particle toxicity [53,56,59]. Therefore, the increased expressions of *IL-1a*, *IL-6*, and *IL-8* within the present study indicate a pro-inflammatory response involving both NLRP3 inflammasome and pro-inflammatory transcription factor activation by CuO NP. This response is more pronounced in the co-culture since *IL-1a* and *IL-6* were only induced in this cell model, consistent with elevated IL-6 protein levels after treatment of a co-culture with A549 and dTHP-1 cells with quartz and silica particles [11,14].

## 4. Materials and Methods

All chemicals (p.a. grade), cell culture medium, and supplements were obtained either from Carl Roth GmbH (Karlsruhe, Germany) or Sigma-Aldrich Chemie GmbH (Taufkirchen, Germany) with the exception of fetal bovine serum (FBS), which was bought at Thermo Fisher Scientific GmbH (Dreieich, Germany). Cell culture dishes and flasks, reaction tubes, PCR tubes, and other consumables were purchased from Sarstedt (Nuembrecht, Germany). CuO NP were kindly provided by Dr. Wendel Wohlleben (BASF SE, Germany) in the course of the BMBF-funded project *MetalSafety* and originally synthesized and obtained from by PlasmaChem (Berlin, Germany). This particle species was also previously examined in the EU FP7-Project SUN, including oral and inhalation in vivo studies [28,60]. Primer pairs were synthesized by Eurofins (Ebersberg, Germany). DNA suspension buffer, PCR certified water, and TE buffer were obtained from Teknova (Hollister, USA). The 2× Assay Loading Reagent, 20× DNA Binding Dye, and IFCs were purchased from Fluidigm (San Francisco, USA). The 2× SsoFast EvaGreen Supermix was provided by Bio-Rad (Munich, Germany), and the 2× TaqMan PreAmp Master Mix was bought from Applied Biosystems (Darmstadt, Germany). Exonuclease I was obtained from New England Biolabs (Frankfurt am Main, Germany).

### 4.1. Cell Culture

Human adenocacinoma cell line A549 (ATCC CCL-185) was kindly provided by Dr. Roel Schins (Leibniz Research Institute for Environmental Medicine, Düsseldorf, Germany). A549 cells were cultured as monolayer in RPMI-1640 supplemented with 10% FBS, 100 U/mL penicillin, and 100 µg/mL streptomycin at 37 °C in a humidified atmosphere of 5% CO_2_ in air (HeraSafe, Thermo Scientific, Langenselbold, Germany). For monoculture experiments, 172,000 cells/cm^2^ were seeded in 24-well plates and grown to confluency for 24 h prior to NP incubation.

THP-1 cells (human peripheral blood monocytes, ATCC TIB-202) were kindly provided by Dr. Richard Gminski (Albert-Ludwig-University Freiburg, Department of Environmental Health Sciences and Hygiene, Freiburg, Germany). THP-1 cells were cultured in suspensions with cell densities below 1 × 10^6^ cells/mL in supplemented RPMI-1640 (see above). Prior to seeding for co-culture experiments, 0.93 × 10^5^ cells/cm^2^ were seeded in cell culture dishes and differentiated for four days with 30 ng/mL phorbol 12-myristate 13-acetate (PMA, diluted in DMSO). After four days, cell culture medium was changed to supplemented RPMI-1640 without PMA, and the differentiated THP-1 cells (dTHP-1) were cultured for further 3–5 days [61]. For co-culture experiments, 172,000 A549 cells/cm^2^ were seeded and incubated for four hours before fresh cell culture medium containing 35,000 dTHP-1 cells/cm^2^ was added. Co-culture was grown to confluency for a further 20 h. These cell numbers were selected to ensure a ratio of 1 macrophage per 10 epithelial cells at the time of exposure (24 h after seeding) to simulate in vivo conditions [29,62]. For all experiments with dTHP-1 cells, accutase instead of trypsine was used to detach adherent cells.

### 4.2. Nanoparticle Preparation and Characterization

The nanoparticles were dispersed freshly according to the latest NANOGENTOX protocol at a concentration of 2.56 mg/mL in 0.05% BSA in bidest H_2_O [63]. Briefly, 15.36 mg were pre-wetted with 30 µL 97% ethanol before adding 5.970 mL 0.05% BSA in bidest. H_2_O generating a stock solution of 2.56 mg/mL. Subsequent sonification was performed using a Branson Analog Sonifier 450 (Brookfield, CT, USA) for 13:25 min at 10% amplitude (7179 J). After sonification, the stock solution was diluted in supplemented RPMI-1640 to achieve the respective incubation concentrations.

Hydrodynamic particle diameter and ζ-potential were determined by dynamic light scattering (DLS) using a Zetasizer NANO ZS (Malvern, Herrenberg, Germany) equipped with a 532 nm laser. Analysis was performed with a 100 µg/mL dispersion in supplemented RPMI-1640. Hydrodynamic diameter was obtained as Z-average in 10 replicates of three independent experiments. Measurement conditions were optimized automatically by the Zetasizer Nano ZS Dispersion Technology Software (v6.20). Dispersant properties were considered equal to pure water (R_i_ = 1.33, viscosity = 0.8872 cP) for CuO R_i_ = 2.58 [18] was used.

For transmission electron microscopy (TEM), the dispersion was applied on a TEM Grid (Plano GmbH, Germany) and subsequently analyzed using a Philips CM200 at the Laboratory for Electron Microscopy at Karlsruhe Institute of Technology. Primary particle size was determined as means of 2179 particles using the software ImageJ.

CuO NP were analyzed for endotoxins using Pierce LAL Chromogenic Endotoxin Quantification Kit (Thermo Fisher Scientific, Dreieich, Germany) using the supernatant of a 2.56 mg/mL stock solution in water. Detection limit of the applied kit was given in the manufacturer’s instructions as 0.1 EU/mL.

Solubility studies were performed by diluting the previously described CuO NP stock solution to a concentration of 100 µg/mL in RPMI (10% FBS) and incubating the solution for 24 h at 37 °C while shaking at 150 rpm in a centrifuge tube. After incubation, the solution was centrifuged at 3000× *g* for 1 h followed by a second centrifugation step of the supernatants at 16,000× *g* for 1 h. Subsequently, 2 mL of each supernatant was collected and centrifuged again at 16,000× *g* for 1 h. Residues of particles in the supernatant were precluded using dynamic light scattering. One milliliter of the supernatant was heated stepwise to 95 °C until full dryness and subsequently oxidized with a 1:1 mixture of HNO_3_ (69%)/H_2_O_2_ (31%) (*v*/*v*) by stepwise heating to 95 °C. The residue was solubilized in 1 mL HNO_3_ (0.2%) and copper content was measured by GF-AAS (Pinaccle 900 T, Perkin Elmer, Rodgau, Germany).

### 4.3. Cytotoxicity Assays

For relative cell count (RCC), cells were incubated with CuO NP for 24 h in 24-well plates and subsequently either trypsinized for 2 min at 37 °C (A549) or detached using accutase for 15 min at 37 °C (A549+dTHP-1, dTHP-1). After detachment, cells were suspended in supplemented RPMI-1640 and counted using a CASY Cell Counter (OLS OMNI Life Science GmbH, Bremen, Germany).

For analyzing the ATP content, CellTiter-Glo^®^ Luminescent Cell Viability Assay Kit (Promega GmbH, Walldorf, Germany) was applied. Cell cultivation and incubation were performed in 96-well plates. Briefly, 6.4 × 10^4^ A549 cells per well were seeded for monoculture. For co-culture 9,600 dTHP-1 cells were added per well following the protocol stated above. The cells were incubated with CuO NP or 500 nM staurosporine (positive control) for 24 h. After removing the incubation medium, 100 μL of fresh medium were added to the wells, the plate was equilibrated for 30 min at room temperature, and 100 μL of CellTiter-Glo^®^ reagent was added. Chemiluminescence was measured on the Infinite^®^ 200 Pro microplate reader (Tecan Group Ltd., Männedorf, Switzerland) after short-term orbital shaking and a further 10 min to stabilize the signal.

### 4.4. High-Throughput RT-qPCR

Transcriptional toxicity profiles were obtained using quantitative high-throughput RT-qPCR with Fluidigm dynamic arrays on the BioMark system as described previously [25], but applying a gene set supplemented with genes coding for fibrosis and inflammatory markers as described above. Data were processed using Fluidigm Real-Time PCR Analysis as well as Genex software. Five reference genes (*ACT*, *B2M*, *GAPDH*, *GUSB*, and *HPRT1*) were used for normalization. Expression changes of the respective genes were displayed as fold change compared to untreated controls by calculating relative quantities corresponding to the ΔΔCq method [64].

### 4.5. Statistics

Within this study, the means of at least three independently performed experiments are displayed, if not stated otherwise. Differences between untreated controls and nanoparticle treatments were analyzed by one-way ANOVA followed by Dunnet’s post-hoc test for multiple comparisons. When comparing one dose in between the cell culture models, unpaired *t*-test was used. Results were considered statistically significant at *p* ≤ 0.05.

## 5. Conclusions

In summary, no pronounced differences in CuO NP-induced cytotoxicity between the two cell models were observed. Furthermore, both cell cultures revealed transcriptional toxicity profiles upon CuO NP treatment, which is consistent with previous studies. However, a cell culture-dependent difference was observed regarding the inflammatory response after CuO NP incubation. While mono-cultured A549 cells were limited to a transcriptionally activated *IL-8*, the co-culture system revealed an involvement of NLRP3 inflammasome by *IL-1a* up-regulation and extended the transcriptional activated pro-inflammatory response by enhanced *IL-6* expression. This supports the use of co-culture models to obtain comprehensive toxicity profiles of nanomaterials and indicates the advantage and suitability of a simple advanced in vitro model of A549 + dTHP-1 cells for high-throughput toxicity screening of nanomaterials. Nevertheless, as is the case with all in vitro systems, one has to be aware of potential limitations in the cell lines applied, and in the case of A549 cells, the deregulated Nrf2 response, resulting in a limited antioxidant defense. Here, the application of Beas-2B cells could possibly improve the prediction towards in vivo studies.

## Figures and Tables

**Table 1:** Summary of the nanoparticle characterization.

d_primary_ (nm)	d_hyd_ (nm)	PDI	ζ-Potential (mV)	SSA (m^2^/g)	Solubility in RPMI ^2^
17.1 ± 0.4	175 ± 44	0.49 ± 0.045	−14.8 ± 0.2	47 ^1^	23 ± 12%

The determination of the hydrodynamic diameter (d_hyd_), ζ-potential, and solubility in cell culture media after 24 h were performed in a suspension of 100 µg/mL in cell culture medium (RPMI, supplemented with 10% fetal bovine serum (FBS)). d_primary_: primary particle size, SSA: specific surface area, PDI: polydispersity index. ^1^ From Sustainable Nanotechnolgies (SUN) project [27]. ^2^ Supplemented with 10% FBS.

## Data Availability

The data presented in this study are available on request from the first (MH) and corresponding author (AH) for researchers of academic institutes who meet the criteria for access to the confidential data.

## Supplementary Materials

Supplementary Materials can be found at https://www.mdpi.com/article/10.3390/ijms22095044/s1.

## Abbreviations

BSA	Bovine serum albumin
DLS	Dynamic light scattering
dTHP-1	Differentiated monocytic THP-1 cells to macrophage-like cells
EMT	Epithelial–mesenchymal transition
FBS	Fetal bovin serum
GF-AAS	Graphite Furnace Atomic Absorption Spectrometry
HT RT qPCR	High-throughput RT-qPCR
MoA	Mode of Action
NP	Nanoparticle
PDI	Polydispersity Index
RCC	Relative Cell Count
ROS	Reactive oxygen species
TEM	Transmission electron microscopy
XPS	X-ray photoelectron spectroscopy
XRD	X-ray diffraction
