# Supplementary material for: Impact of Differentiated Macrophage-Like Cells on the Transcriptional Toxicity Profile of CuO Nanoparticles in Co-Cultured Lung Epithelial Cells
Source: Int J Mol Sci. 2021 May 10;22(9):5044. doi: 10.3390/ijms22095044 (PMC8126233; doi:10.3390/ijms22095044)
Supplement: Supplementary file 1 [file ijms-22-05044-s001.zip › ijms-1186294-supplementary.pdf]

International Journal of *Molecular Sciences*

MDPI

Supplementary files

Impact of differentiated macrophage-like cells on the transcriptional toxicity profile of CuO nanoparticles in co-cultured lung epithelial cells

Matthias Hufnagel [1], Ronja Neuberger [1], Johanna Wall [1], Martin Link [1], Alexandra Friesen[1] and Andrea Hartwig [1,*]

[1] Department of Food Chemistry and Toxicology, Institute of Applied Biosciences, Faculty of Chemistry and Biosciences, Karlsruhe Institute of Technology (KIT)

* Correspondence: andrea.hartwig@kit.edu

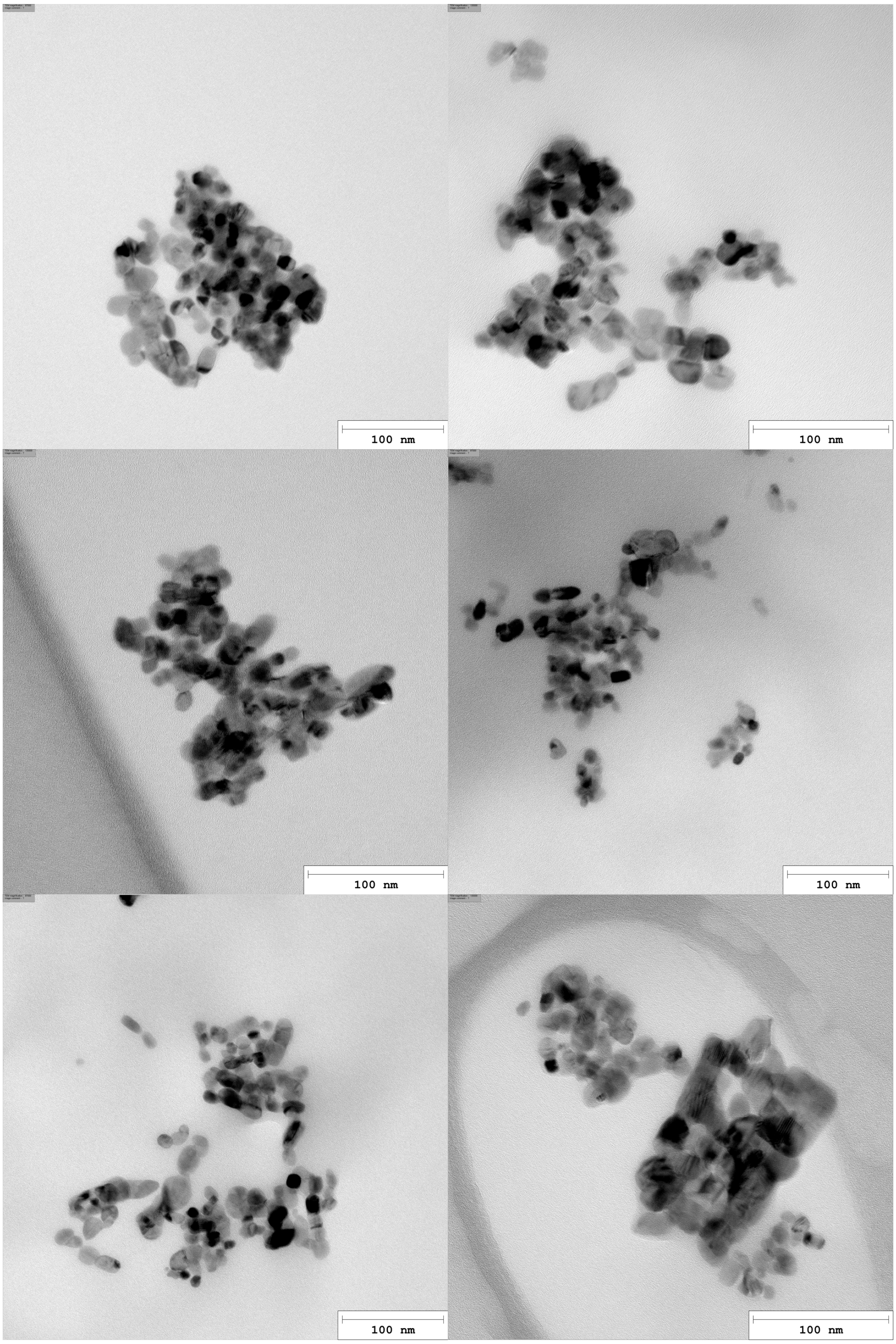

Supplementary Figure S1. Representative TEM images of CuO NP at 2.56 mg/mL in 0.05 % BSA after dispersion according to the described dispersion protocol.

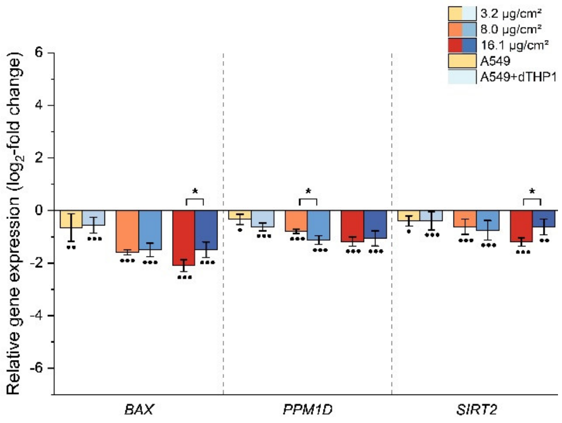

Supplementary Figure S2. Impact of CuO NP on genes related to apoptosis in A549 monoculture (A549) and co-culture (A549+dTHP-1) after 24 h incubation. Depicted is the log_2-fold change of at least three independently conducted experiments ± SD. Significantly different from negative controls: ● ≤ 0.05 (ANOVA-Dunnett's *t*-test); significantly different between cell models: * ≤ 0.05 (unpaired *t*-test).

Supplementary Table S1. Extended primer set for inflammatory response and fibrosis.

Gene	Name of the coding protein	Gene ID	RefSeq		Sequence (5′ → 3′)	Position RefSeq (nt)	Position exon	Amplicon-size [bp]	T_m Amplicon [°C]	Isoform hits
CCL22	C-C motif chemokine 22 (CCL22)	6367	NM_002990.5	fwd:	CGTCTGCTGCCGTGATTAC	136	2	191	81	NM_002990.5
				rev:	AGTAGGCTCTTCATTGGCTCAG	326	3			
PTGS2	Prostaglandin G/H synthase 2 (PGH2)	5743	NM_000963.4	fwd:	CCGAGGTGTATGTATGAGTGTGG	85	2	136	76	NM_000963.4
				rev:	TGTGTTTGGAGTGGGTTTCAGA	220	3			
IL-1a	Interleukin-1 alpha (IL-1 alpha)	3552	NM_000575.5	fwd:	GAATGACGCCCTCAATCAAAGT	467	5	203	79	NM_000575.5;
				rev:	GCAGCACTGGTTGGTCTTC	669	6			NM_001371554.1
IL-1b	Interleukin-1 beta (IL-1 beta)	3553	NM_000576.2	fwd:	GAGGCACAAGGCACAACA	16	3	186	82	NM_000576.2
				rev:	CCTGGAAGGAGCACTTCATCT	199	4			
IL-6	Interleukin-6 (IL-6)	3569	NM_000600.4	fwd:	CCAATCTGGATTCAATGAGGAGAC	427	3-4	158	80	NM_001318095.2
				rev:	GCAGGAACTGGATCAGGACTT	584	4			NM_001371096.1
TNF	Tumor necrosis factor (TNF-a)	7124	NM_000594.4	fwd:	ACACCATGAGCACTGAAAGC	173	1	225	87	NM_000594.4
				rev:	CCAGAGGGCTGATTAGAGAGA	397	2			
ACTA2	actin, aortic smooth muscle (ACTA2)	59	NM_001141945.2	fwd:	GACCTTTGGCTTGGCTTGT	59	1	117	84	NM_001141945.2;
				rev:	GGTGCGGACAGGAATTGAAG	175	1			NM_001320855.1;
										NM_001613.4
COL1A1	collagen alpha-1(I) chain (CO1A1)	1277	NM_000088.3	fwd:	GCCAAGACGAAGACATCCC	215	1-2	235	88	NM_000088.3
				rev:	GTGGTTTCTTGGTCGGTGG	499	3			
CTNNB1	catenin beta-1 (CTNB1)	1499	NM_001098209.2	fwd:	CTGAGGAAGAGGATGTGGATACC	369	3	199	81	NM_001098209.2;
				rev:	TGAGCAGCATCAAACTGTGTAGA	567	4			NM_001098210.2;
										NM_001330729.2;
										NM_001904.4
FN1	Fibronectin (FN)	2335	NM_001306129.1	fwd:	CGGTGGCTGTCAGTCAAAG	393	1	128	81	NM_001306129.1;
				rev:	ACCTCGGCTTCCTCCATAAC	520	2			NM_001306130.2;
										NM_001306131.2;
										NM_001306132.2;
										NM_001365517.2;

										NM_001365518.2;<br>NM_001365519.2;<br>NM_001365520.2;<br>NM_001365521.2;<br>NM_001365522.2;<br>NM_001365523.2;<br>NM_001365524.2;<br>NM_002026.4;<br>NM_054034.3;<br>NM_212474.3;<br>NM_212476.3;<br>NM_212478.3;<br>NM_212482.4
SPP1	Osteopontin (OPN)	6696	NM_000582.2	fwd:<br>rev:	TGAATCTGATGAACTGGTCACTGA<br>TGATGTCCTCGTCTGTAGCA	504<br>691	5<br>6	188	82	NM_000582.2;<br>NM_001040058.2;<br>NM_001040060.2;<br>NM_001251829.2;<br>NM_001251830.2
PDGFA	Platelet-derived growth factor subunit A (PDG-FA)	5154	NM_002607.5	fwd:<br>rev:	CGATGAGGACCTTGGCTTG<br>CTTCTCGGGCACATGCTTAG	842<br>1074	1<br>6	233	87	NM_002607.5;<br>NM_033023.5
TGFB1	Transforming growth factor beta-1 (TGFB1)	7040	NM_000660.5	fwd:<br>rev:	GGAAACCCACAACGAAATCTATGA<br>GCTGAGGTATCGCCAGGAAT	1181<br>1388	1-2<br>3	208	84	NM_000660.5
TIMP1	Metalloproteinase inhibitor 1 (TIMP1)	7076	NM_003254.3	fwd:<br>rev:	GATACTTCCACAGGTCCCACAAC<br>GGTCCGTCCACAAGCAATGA	328<br>564	4<br>6	237	85	NM_003254.3
VIM	Vimetin (VIME)	7431	NM_003380.5	fwd:<br>rev:	AAGACGGTTGAAACTAGAGATGGA<br>TGTAGGAGTGTCGGTTGTTAAGAA	1764<br>1995	9<br>10	232	79	NM_003380.5

All other used primers of the used gene set were describe in [22], with exception of *ABCB1, ABCC1, ADH1B, ALDH1A1, GSTP1, NAT1, SULT1A1, UGT1A, SEPP1, TP53, AXIN2, BCL2L1, ERCC2, POLB, OGG1,* and *XRCC5* which were removed from the gene set to open space for inflammatory and fibrotic marker genes.

Supplementary Table S2. Log2-fold changes in gene expression and p-values of untreated co-culture controls compared to A549 monoculture.

Gene name	Log2-fold change	p-value	Gene name	Log2-fold change	p-value
CYP1A1	-0.38	0.001	*BCL2*	0.00	0.992
AHR	-0.33	0.178	*BTRC*	-0.26	0.002
NQO1	-0.48	0.000	*CCND1*	-0.50	0.000
CCL22	5.00	0.000	*CDKN1A*	0.49	0.000
PTGS/COX2	-0.22	0.223	*CDKN1B*	-0.29	0.000
IL-1b	4.18	0.000	*E2F1*	-0.80	0.000
IL-6	0.54	0.262	*EGFR*	-0.12	0.055
IL-8	0.15	0.455	*JUN*	0.27	0.022
ACTA2	0.33	0.019	*MDM2*	-0.25	0.004
CTNNB1	-0.10	0.043	*MYC*	-0.18	0.027
FN1	0.17	0.237	*PLK3*	0.23	0.173
SPP1	0.36	0.025	*PMAIP1*	-0.11	0.025
PDGFA	0.12	0.209	*PPM1D*	-0.12	0.111
TGFB1	0.07	0.284	*SIRT2*	-0.13	0.579
TIMP1	-0.23	0.010	*TNFRSF10B*	-0.32	0.000
VIM	-0.06	0.506	*VEGFA*	0.03	0.864
FTH1	0.21	0.309	*XIAP*	-0.11	0.255
MT1X	-0.20	0.016	*APEX1*	-0.19	0.049
MT2A	-0.43	0.001	*ATM*	-0.24	0.000
SLC30A1	-0.14	0.150	*ATR*	-0.48	0.000
TFRC	-0.19	0.138	*BRCA1*	-0.31	0.004
CAT	-0.26	0.000	*BRCA2*	-0.41	0.000
G6PD	-0.59	0.000	*DDB1*	-0.35	0.008
GCLC	-0.30	0.000	*DDB2*	0.03	0.840
GPX1	0.30	0.026	*DDIT3*	0.66	0.002
GPX2	-0.21	0.031	*ERCC1*	-0.24	0.013
GSR	-0.51	0.000	*ERCC4/XPF*	-0.42	0.000
HMOX1	0.61	0.000	*ERCC5/XPG*	-0.33	0.000
HSPA1A	-0.54	0.001	*GADD45A*	0.43	0.003
KEAP1	-0.29	0.004	*LIG1*	-0.50	0.000
MAP3K5	-0.15	0.057	*LIG3*	-0.51	0.000
NFE2L2	-0.17	0.071	*MGMT*	-0.11	0.417
NFKB1	-0.06	0.283	*MLH1*	-0.49	0.000
NFKB2	0.49	0.005	*MSH2*	-0.27	0.000
NFKBIA	0.25	0.275	*PARP1*	-0.40	0.001
PRDX1	-0.33	0.012	*PCNA*	-0.24	0.014
SOD1	-0.31	0.010	*POLD1*	-0.24	0.071
SOD2	0.15	0.196	*POLQ*	-0.49	0.000
TXN	-0.45	0.000	*RAD50*	0.04	0.674
TXNRD1	-0.57	0.000	*RAD51*	-0.41	0.000
APAF1	-0.07	0.467	*RRM2B*	-0.20	0.044
BAX	0.02	0.707	*XPC*	-0.19	0.024

BBC3	0.35	0.089

Supplementary Table S3. X-fold changes in gene expression treated mono- and co-cultures compared to untreated controls. N.q.: not quantifiable.

	A549			A549+dTHP-1		
**Dose (µg/mL)**	**3.2**	**8.0**	**16.1**	**3.2**	**8.0**	**16.1**
AHR	0.2	0.1	0.1	0.4	0.1	0.1
CYP1A1	4.5	6.0	6.2	3.9	7.0	4.9
NQO1	0.5	0.5	0.6	0.6	0.5	0.6
CCL22	1.0	1.8	2.6	0.2	0.1	0.0
PTGS/COX2	0.1	0.1	0.1	0.4	0.1	0.2
IL-1a	n.q.	n.q.	n.q.	7.4	5.6	2.0
IL-1b	0.5	0.6	0.5	0.2	0.2	0.1
IL-6	0.5	0.7	0.6	6.1	6.2	1.7
IL-8	2.6	19.9	50.3	4.0	14.4	37.8
TNF-a	n.q.	n.q.	n.q.	0.5	0.4	0.3
ACTA2	0.2	0.1	0.2	0.5	0.1	0.2
COL1A1	n.q.	n.q.	n.q.	1.1	0.6	1.2
CTNNB1	0.7	0.4	0.3	0.8	0.5	0.4
FN1	0.4	0.5	0.6	0.5	0.4	0.6
SPP1	0.8	0.6	0.5	0.6	0.6	0.7
PDGFA	0.3	0.1	0.0	0.4	0.1	0.1
TGFB1	0.5	0.5	0.5	0.7	0.5	0.5
TIMP1	0.7	1.1	1.2	0.8	1.2	1.6
VIM	0.5	0.4	0.7	0.6	0.5	0.6
FTH1	1.5	1.2	0.8	1.7	1.3	0.9
MT1X	2.3	8.5	8.8	34.0	8.0	7.6
MT2A	1.3	13.2	12.6	25.6	14.7	14.3
SLC30A1	2.1	0.6	0.6	2.4	0.7	0.7
TFRC	0.9	0.2	0.2	1.5	0.4	0.2
CAT	0.5	0.3	0.3	0.7	0.4	0.4
G6PD	0.4	0.5	0.5	0.6	0.5	0.5
GCLC	0.4	0.2	0.1	0.5	0.2	0.2
GPX1	0.7	0.8	0.8	0.5	0.5	0.8
GPX2	0.3	0.2	0.2	0.4	0.2	0.4
GSR	0.9	0.8	0.8	1.1	1.1	0.8
HMOX1	22.6	28.4	23.6	12.1	21.9	21.7
HSPA1A	16.7	57.6	58.5	16.0	68.2	69.8
KEAP1	0.3	0.1	0.1	0.5	0.1	0.1
MAP3K5	0.4	0.1	0.1	0.4	0.1	0.1
NFE2L2	0.5	0.1	0.1	0.6	0.2	0.1
NFKB1	0.8	0.3	0.2	1.0	0.5	0.3

NFKB2	3.0	1.0	0.5	2.8	0.9	0.5
NFKBIA	0.9	0.2	0.0	1.3	0.2	0.0
PRDX1	1.0	1.2	1.3	1.1	1.6	1.4
SOD1	1.0	1.1	1.2	1.1	1.6	1.4
SOD2	1.1	1.3	1.1	2.6	2.7	1.9
TXN	0.8	1.0	1.3	1.0	1.6	1.6
TXNRD1	0.8	0.8	1.0	1.1	1.0	1.2
APAF1	0.7	0.3	0.1	0.7	0.3	0.2
BAX	0.6	0.3	0.2	0.7	0.4	0.4
BBC3	0.2	0.1	0.1	0.4	0.1	0.1
BCL2	0.5	0.2	0.3	0.4	0.2	0.2
BTRC	0.8	0.7	0.6	0.8	0.8	0.7
CCND1	0.5	0.4	0.4	0.6	0.4	0.3
CDKN1A	1.0	0.8	0.5	1.0	0.7	0.5
CDKN1B	0.2	0.1	0.0	0.3	0.1	0.1
CDKN2B	n.q.	n.q.	n.q.	n.q.	n.q.	n.q.
E2F1	0.9	0.5	0.6	1.3	0.5	0.6
EGFR	0.6	0.4	0.4	0.9	0.4	0.4
JUN	1.2	2.3	3.5	1.0	1.4	2.6
MDM2	0.5	0.3	0.3	0.6	0.4	0.3
MYC	0.3	0.1	0.0	0.4	0.1	0.0
PLK3	0.6	0.2	0.1	0.7	0.2	0.2
PMAIP1	0.9	0.8	0.7	1.1	0.9	0.9
PPM1D	0.8	0.6	0.4	0.6	0.5	0.5
SIRT2	0.8	0.7	0.4	0.8	0.6	0.6
TNFRSF10B	1.1	0.7	0.6	1.4	0.9	0.9
VEGFA	1.4	0.6	0.4	1.5	0.6	0.5
XIAP	0.7	0.5	0.6	0.7	0.7	0.6
APEX1	0.7	0.3	0.4	0.8	0.4	0.5
ATM	0.5	0.3	0.3	0.7	0.3	0.4
ATR	0.6	0.2	0.1	0.7	0.3	0.1
BRCA1	0.8	0.2	0.1	1.0	0.3	0.2
BRCA2	0.9	0.2	0.1	1.2	0.4	0.2
DDB1	0.6	0.5	0.5	0.8	0.6	0.5
DDB2	0.5	0.1	0.1	0.7	0.1	0.1
DDIT3	6.0	14.9	18.2	4.6	7.2	22.5
ERCC1	0.5	0.5	0.6	0.6	0.5	0.7
ERCC4/XPF	0.3	0.1	0.0	0.4	0.1	0.1
ERCC5/XPG	0.3	0.2	0.1	0.4	0.2	0.1
GADD45A	2.5	3.3	3.1	2.3	3.5	5.3
LIG1	0.6	0.3	0.2	0.8	0.3	0.3
LIG3	0.4	0.2	0.2	0.5	0.3	0.2
MGMT	0.6	0.9	1.0	0.6	0.7	1.1
MLH1	0.7	0.3	0.2	10	0.4	0.3
MSH2	0.7	0.2	0.1	0.8	0.2	0.2
PARP1	0.7	0.4	0.4	0.8	0.4	0.4
PCNA	0.8	0.1	0.0	0.9	0.2	0.1

POLD1	0.9	0.3	0.2	1.0	0.4	0.3
POLQ	0.6	0.3	0.3	0.9	0.3	0.4
RAD50	0.4	0.2	0.2	0.5	0.2	0.3
RAD51	1.4	0.2	0.1	1.7	0.4	0.1
RRM2B	0.5	0.3	0.3	0.7	0.4	0.3
XPA	n.q.	n.q.	n.q.	n.q.	n.q.	n.q.
XPC	0.3	0.1	0.1	0.5	0.1	0.1
